# Alpha Linolenic and Stearic Acids Modulate Genes Related to Viral Entry and Inflammatory Response in THP‐1 Derived Macrophages Exposed to SARS‐CoV‐2

**DOI:** 10.1002/fsn3.70529

**Published:** 2025-09-26

**Authors:** Aline Rosa Maia, Bruna Rafaela dos Santos Silva, Pierina Lorencini Parise, Camila Lopes Simeoni, Luana Satelis Meira, José Luiz Proença Módena, Eliana Pereira Araújo, Licio Augusto Velloso, Gabriel Forato Anhê, Joseane Morari

**Affiliations:** ^1^ Obesity and Comorbidities Research Center Institute of Biology, Universidade Estadual de Campinas (UNICAMP) Campinas Sao Paulo Brazil; ^2^ School of Nursing Universidade Estadual de Campinas (UNICAMP) Campinas Brazil; ^3^ Department of Genetics, Evolution, Microbiology and Immunology Institute of Biology, Universidade Estadual de Campinas (UNICAMP) Campinas Sao Paulo Brazil; ^4^ Department of Pharmacology School of Medical Sciences, Universidade Estadual de Campinas (UNICAMP) Campinas Sao Paulo Brazil

**Keywords:** inflammation, macrophage, NEFAs, obesity, SARS‐CoV‐2

## Abstract

Obesity plays a role in the poor prognosis of COVID‐19. The study was designed to elucidate if alpha linolenic (Ala) and stearic (Est) acids could modulate the inflammatory response to SARS‐CoV‐2. Firstly, scRNA‐seq data analysis from COVID‐19 patients revealed that major changes in the expression of genes related to virus entry to the cell and inflammatory response were found in macrophages rather than in alveolar epithelial cells. In vitro experiments were performed with Beas2B (human bronchial epithelial cells) and THP‐1 (human monocytes/macrophages) cells to verify the putative effects of Ala and Est in the response to either Spike protein (S3) or the full SARS‐CoV‐2. Both Ala and Est exacerbated the increase in the expression of genes related to virus entry into the cell and pro‐inflammatory cytokines in THP‐1 derived macrophages. These data suggest that NEFAs may play a role in the poor prognosis seen in individuals with obesity affected by COVID‐19.

## Graphical abstract

Obesity disease associates with subclinical inflammation and excess of non‐esterified fatty acids (NEFAs). In the presence of Spike protein (S3) and SARS‐CoV‐2, NEFAs modulates genes related to the viral entry and the inflammatory response in THP‐1 derived macrophages. Excess of NEFAs may negatively impact the prognosis in individuals with obesity affected by COVID‐19.

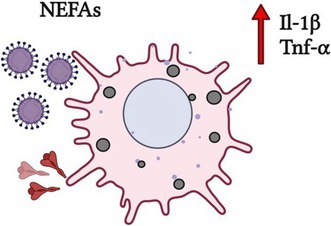

## 1 Introduction

The non‐segmented enveloped severe acute respiratory syndrome coronavirus 2 (SARS‐CoV‐2) is a sense positive single stranded RNA virus (ssRNA+) described in China in December 2019 as the agent responsible for the COVID‐19 pandemic. The virus is a β‐coronavirus that shares similarities with the previously described coronaviruses SARS‐CoV and MERS‐CoV. SARS‐CoV‐2 is formed by four main structural proteins: glycoprotein S from the spike, glycoprotein E from the small envelope, glycoprotein M from the membrane, and the nucleocapsid protein N (López‐Collazo et al. 2020).

The spike protein exhibits high affinity for human ACE2 (Zhang et al. 2018). Spike suffers the cleavage by host furins and serine protease TMPRSS2 enabling viral entry into the cells (Mehta et al. 2020). In cells that express low levels of ACE2 at the membrane, phosphatidylserine receptors like AXL bind to phosphatidylserine residue of viral spike protein to mediate virus internalization through an endosomal route (Bohan et al. 2021).

Infection with SARS‐CoV‐2 can evolve to asymptomatic, moderate or severe disease. Severely affected patients usually present fever, dry cough, dyspnea and fatigue, which are commonly associated with the development of pneumonia and acute respiratory distress syndrome (ARDS) (Zhou, Yu, et al. 2020; Borobia et al. 2020; Zhou, Yang, et al. 2020; Chen et al. 2020; Li et al. 2020). COVID‐19 infection triggers a cytokine storm characterized by imbalanced and uncontrolled cytokine response especially in critically ill patients, who also present lymphopenia. The macrophages resident in tissues, including lungs, release large amounts of pro‐inflammatory cytokines like IL‐1β, IL‐6, TNFɑ and IL‐8 (Eastin and Eastin 2020; Qin et al. 2020).

Several comorbidities such as obesity play a pivotal role in poor prognosis of COVID‐19, increasing the risk for hospitalization and mortality (Thoppil et al. 2022). The precise mechanism underlying the poor prognosis in obese COVID‐19 patients however, remains unknown. ACE2 is highly expressed in adipocytes and therefore it has been proposed that the adipose tissue serve as a reservoir for the virus. Obese individuals would therefore contain a source for a long term release of SARS‐CoV‐2 particles to blood stream, boosting the inflammatory response to the virus and its clinical symptoms (Ryan and Caplice 2020; Saccon et al. 2022).

Besides the increase in adiposity, obesity is also hallmarked by complex biochemical changes in the internal milieu. The adipose tissue itself can act as an endocrine organ that releases a myriad of cytokines that potentially contribute to cytokine storm, such as IL‐1β, IL‐6, IL‐8, and TNF‐α, and specific hormones such as adiponectin and leptin to the circulation (Ouchi et al. 2011; Li et al. 2018). Increased adipose tissue mass also serves as a source of non‐esterified fatty acids (NEFA) that are released to the circulation, contributing to obesity‐associated dyslipidemia. However, it is not clear if NEFAs can modulate the virus entry or the inflammatory response of macrophages or pulmonary epithelial cells to SARS‐CoV‐2 (Opie and Walfish 1963; Porsche et al. 2021).

## 2 Material and Methods

### 2.1 Single‐Cell RNA‐Sequencing (scRNA‐Seq) Analysis

For scRNA‐seq analysis, datasets from MERTK+F13A1+ pulmonary macrophages, related to inflammation resolution (Cai et al. 2018), and AT2 SFTPC+ alveolar epithelial cells, known for their expression of surfactant protein C (O'Reilly 2017), were retrieved from Gene Expression Omnibus (GEO) with accession number GSE171668.

Matrices were arrayed and analyzed according to COVID‐19 status (positive or negative), and the presence of obesity (BMI > 30). The raw data with the .*h5* format were loaded for analysis through the toolkit Scanpy 1.9.3 in Python igraph 0.10.4. The quality control was performed by excluding samples containing less than 200 genes and more than 1500 genes, or if mitochondrial genes were > 15% of the total. The highly variable genes were identified by using the *pp.highly_variable_genes* function for the downstream principal component analysis (PCA) (Luecken and Theis 2019). Clustering was performed by the Leiden graph‐clustering method and the top 50 principal components were chosen (Traag et al. 2019). The cell type identity mtt was manually annotated, and the differentially expressed genes (DEG) for a cluster and all remaining cells were calculated by the two‐sided Wilcoxon rank‐sum test (Xun and Garmire 2019). The marker genes were then ranked by their log fold change of expression in particular cell types.

### 2.2 Experimental Design

#### 2.2.1 Free Fatty Acid Dilution

The alpha linolenic acid (Ala, 18:3 ω‐3) was first diluted in alcohol 99% P.A. to 0.5 M (stock solution). To prepare the working solution, the stock solution was first heated to 60°C for 1 min and diluted in the culture medium at 37°C to a final concentration of 100 μM.

The stearic acid (Est, 18:0) was first diluted in water to 0.5 M (stock solution) and heated to 60°C for 2 min. To prepare the working solution, the stock solution was first heated to 60°C for 1 min and diluted in the culture medium at 37°C to a final concentration of 100 μM (Regina‐Ferreira et al. 2024; Ye et al. 2016; Tse and Belsham 2018).

#### 2.2.2 SARS‐CoV‐2 and Recombinant Spike Protein

SARS‐Cov‐2 of Delta (δ) strain (BeH823339, GenBank KU729217), were originally isolated from nasopharyngeal swab samples to yield stocks that were produced according to previous description (Souza et al. 2021) and used in previous studies (Saccon et al. 2022; Barreto et al. 2023; Silva et al. 2024). This variant was kindly donated in 2021 by the Emerging Viruses Laboratory of the State University of Campinas (UNICAMP) and was the most prevalent variant at the time we started our experiments. All experiments involving the virus were conducted in a Biosafety Level 3 (BSL3) located at the Emerging Viruses Laboratory of the University of Campinas (UNICAMP).

The recombinant SARS‐CoV‐2 Spike protein (kindly provided by the Cell Culture Engineering Laboratory, COPPE, from Federal University of Rio de Janeiro, Rio de Janeiro, Brazil) was purified by affinity chromatography from HEK293–3F6 cells (NRC Canada) expressing the sequence encoding the ectodomain (amino acids 1–1208) of the spike protein in the prefusion conformation. The addition of two proline amino acids to the C‐terminal of S2 fusion machinery was adopted as an effective strategy for stabilization. The resulting protein was a trimeric version of the complete SARS‐CoV‐2 spike protein ectodomain comprising both the S1 domain, responsible for receptor binding, and S2, responsible for cell membrane fusion with pre‐fusion stabilized conformation (Alvim et al. 2022). This protein was successfully used in the immunization of horses to produce hyperimmune globulins against SARS‐CoV‐2 (Cunha et al. 2021) and other studies used this spike protein to induce immune responses to SARS‐CoV‐2 (Sousa et al. 2023; da Silva et al. 2024; Melgaço et al. 2021).

#### 2.2.3 Cell Culture

Cells were kept in a 37°C humidified incubator aired with 5% CO_2_. Human bronchial epithelial cells Beas‐2B were cultured in RPMI 1640 medium (Gibco—Thermo Fischer Scientific) with 5% FBS, 1:1000 penicillin and streptomycin, and gentamicin. Upon reaching 80% of confluence, Beas‐2B cells were plated in 24‐well plates (1 × 10^5^/well).

Human monocyte cells THP‐1 (ATCC) were cultured in RPMI 1640 medium containing 4,5 g/L of glucose (Gibco—Thermo Fischer Scientific) and 10% FBS. Confluent THP‐1 monocytes were plated in 24‐well plates (4 × 10^5^/well) and differentiated into macrophages after overnight incubation with 1 μg/mL Phorbol 12‐myristate 13‐acetate (PMA) (Sigma Aldrich) followed by a 4‐h resting in PMA‐free medium.

The Beas‐2B cells and the differentiated macrophage THP‐1 cells were incubated with the SARS‐Cov‐2 virus (MOI = 0.1) for 30 min under agitation (Codo et al. 2020; Carmo et al. 2024). After this interval, only THP‐1 cells had the medium replaced by a virus‐free medium containing either 100 μM alpha‐linolenic acid (Ala), 100 μM stearic acid (Est) or their respective vehicles (control group). Cells were kept with this treatment for 24 h. No alterations in cell viability were observed after the incubation with the fatty acids (Figure S1). In additional experiments, differentiated THP‐1 cells were also incubated for 24 h with the recombinant spike protein (5 μg/mL) in the presence of either Ala, Est, or vehicles.

### 2.3 RNA Extraction and Quantitative Polymerase Chain Reaction (qPCR)

After treatments, cells were harvested in 300 μL of Trizol Reagent (Invitrogen—Thermo Fisher Scientific) for total RNA extraction followed by quantification by spectrophotometry. 1250 ηg of RNA of each sample was reversely transcribed into complementary DNA (cDNA) in a thermal cycler using the HighCapacity cDNA Reverse Transcription kit (Applied Biosystems Foster City, CA). Gene expression analyses were carried out in a StepOne Real‐Time PCR System (Applied Biosystems Foster City, CA) using LuminoCt SYBR Green qPCR ReadyMix (Cat No. L6544, Sigma‐Aldrich) or LuminoCt qPCR ReadyMix (Cat No. L6669, Sigma‐Aldrich). The pre‐designed primers (probe included or not included) were purchased from Applied Biosystems and Integrated DNA Technologies (Table S1). The sequences of the primers used with the LuminoCt SYBR Green qPCR ReadyMix were previously described by Assis et al. (2022) (Table S2). Each PCR contained 20 ηg of cDNA, 0.25 μL of ultrapure water and 0.25 μL of primers (forward and reverse). The Peptidylprolyl Isomerase A (*Ppia*) gene was used as the reference gene for reactions with LuminoCt qPCR ReadyMix, while Glyceraldehyde‐3‐Phosphate Dehydrogenase (*Gapdh*) gene was used as reference for reactions with LuminoCt SYBR Green qPCR ReadyMix. The data were analyzed with the 2^^‐ddct^ method using the average of the control group set to 1, and other groups were relative to control (Fold Change).

### 2.4 Viral Quantification by RT‐qPCR

The quantification of SARS‐CoV‐2 was performed by RT‐qPCR using the following set of primers: Forward: 5‐ACA GGT ACG TTA ATA GTT AAT AGC GT‐3; Reverse: 5‐ATA TTG CAG CAGTAC GCA TAC GCA CAC A‐3; Probe: 5–6FAM‐ACA CTA GCC ATC CTT ACT GCG CTT CG‐QSY‐3.

Each PCR contained 3 μL (20 ng) of total RNA, 4 μL of qPCRBIO Probe 1‐Step Go (PCRBIOSYSTEMS, London, UK), 1.0 μL of each primer, 0.5 μL of probe, and 0.5 μL of RTaseGo (PCRBIOSYSTEMS, London, UK).

### 2.5 Statistical Analysis

For the scRNA‐seq analysis, The DEGs of patients with COVID‐19 were determined with Scanpy's implementation of the two‐sided Wilcoxon rank‐sum test. The p_val_cutoff = 0.05 and logfc_cutoff = 0.1 were established by the Scanpy utils function. Experimental results were presented as the mean ± standard error (S.E.M).

Data analysis was performed with two‐way analysis of variance (ANOVA) followed by Tukey's post hoc multiple comparisons. The factors were incubation with fatty acids (factor 1) and incubation with SARS‐Cov‐2 or spike recombinant protein (factor 2). All analyses were performed with GraphPad Prism (Graph Pad Software Inc., Version 8.4, San Diego, USA). P values ≤ 0.05 indicated significant differences.

## 3 Results

### 3.1 scRNA‐Seq From Pulmonary Macrophages (MERTK+F13A1+) and Alveolar Epithelial Cells (AT2 SFTPC+) Revealed Differentially Expressed Genes in COVID‐19 Patients

**FIGURE 1:**
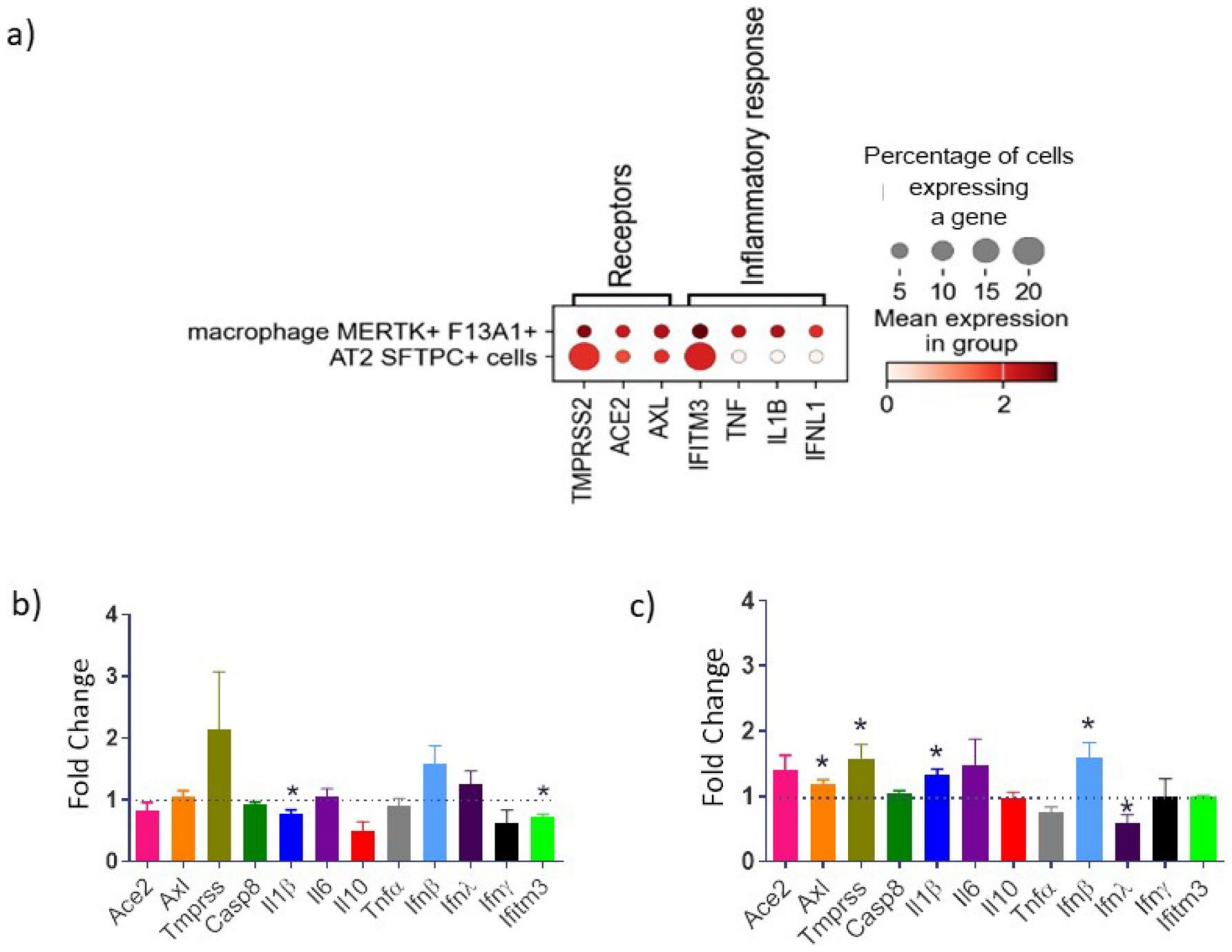
(a) Differentially expressed genes between pulmonary macrophages MERTK^+^F13A1^+^ and alveolar epithelial cells AT2 SFTPC^+^ from coronavirus disease 2019 patients. Experimental results were presented as the mean ± standard error (SEM). (b) Beas 2b cells were incubated with the SARS‐Cov‐2 virus (MOI = 0, 1) SARS‐Cov‐2 for 30 min. (**c)** THP‐1 cells were incubated with the SARS‐Cov‐2 virus (MOI = 0, 1) SARS‐Cov‐2 for 30 min. Beas 2b and THP‐1 data are shown as mean +/− SEM. Bars represent cell exposed to SARS‐CoV‐2 virus in comparison to the dotted line, which expresses values in cells not incubated with the virus, which mean is equose to 1. Data were compared with one‐way ANOVA followed by Tukey post‐test (*p < 0.05); *n* ≥ 3.

Aiming at clarifying the lung cellular type mostly affeected by SARS‐COV‐2 infection, we initially evaluated scRNA‐seq datasets to seek for differential expression of genes of inflammatory responses in pulmonary epithelial cells and macrophages purified from lung tissue of patients with COVID‐19. We found that the mean expression of key genes involved in the SARS‐CoV‐2 infection such as Interferon‐induced transmembrane protein (*Ifitm3*) and Transmembrane Protease Serine 2 (*Tmprss2*) were detected in a higher number of epithelial cells while it was concentrated in a lower fraction of pulmonary macrophages and previous studies are in agreement with productive our findings. In contrast, similar fractions of epithelial cells and macrophages in the lungs express AXL Receptor Tyrosine Kinase (*Axl*), interleukin 1 beta (*Il‐1β*), tumor necrosis factor (*Tnf*) and interferon Lambda (*Ifnλ*), although macrophages display higher mean expression of these genes (Figure 1a). These data support the notion that macrophages, as immune cells, are the main actor in the inflammatory response to SARS‐CoV‐2 infection (Coperchini et al. 2021; Lukassen et al. 2020; Sungnak et al. 2020; Han et al. 2020) (Table S3).

We next performed experiments with the bronchial epithelial cell line (Beas 2B) and the human monocytes (THP‐1) in order to determine if the patterns of expression revealed by analysis of scRNA‐seq could be mimicked when these cells were kept isolated from each other. We evaluated the expression of genes related to the virus pathway/entry into the cells and inflammation after incubation with the SARS‐CoV‐2. The experiments revealed that only Interleukin 1‐beta (*Il‐1β*) and *Ifitm3* were significantly modulated in Beas‐2B cells exposed to the virus (Figure 1b). In contrast, gene expression in THP‐1 cells seems to present a broader response to the virus, having a positive regulation of *Axl, Tmprss, Il‐1β* and *Interferon beta (Ifnβ)* and a downregulation of interferon lambda (*Ifnλ*) (Figure 1c). Importantly, exposure of Beas 2B and THP‐1 to SARS‐Cov2 did not result in productive viral infection, which is in accordance with previously published data using a similar in vitro protocol (Chiok et al. 2023). Accordingly, addition of Est or Ala to the culture medium did not elicit significant productive viral infection (supplementary data—S2).

### 3.2 BMI Impact Gene Expression in COVID19 Patients

**FIGURE 2:**
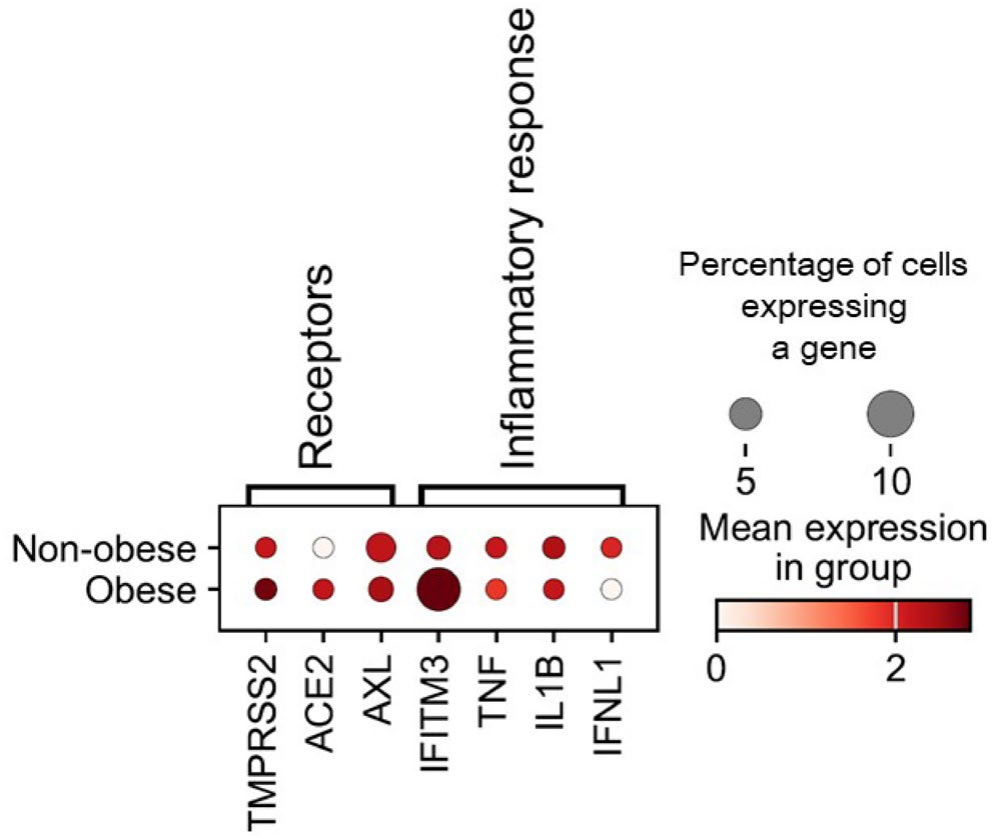
Differentially expressed genes between pulmonary macrophages from obese and non‐obese individuals with coronavirus disease. Experimental results were presented as the mean ± standard error (S.E.M).

In order to check if the genes highly expressed in lung macrophages from COVID‐19 patients are affected by BMI, we rearranged the scRNAseq data comparing the gene expression from obese to those from non‐ obese COVID‐19 patients. The analysis revealed that macrophages of obese COVID‐19 patients have a higher mean expression of *Ace2*, *Tmprss2* and *Ifitm3*. *Axl*, *Tnf‐a* and *Il‐1β* showed the same patterns of expression between obese and non‐obese patients (Figure 2), (Table S4).

We next performed in vitro experiments designed to clarify if increased circulating NEFAs are relevant actors in the obesogenic environment driving the changes in gene expression found in lung macrophages from COVID‐19. We incubated differentiated THP‐1 cells with an unsaturated fatty acid known to elicit anti‐inflammatory responses (alpha linolenic acid [Ala]); or a saturated fatty acid known to stimulate pro‐inflammatory pathways (stearic acid [Est]).

### 3.3 Alpha Linolenic Acid and Stearic Acid Increase the Expression of Genes Related to Viral Entry and Inflammatory Response in THP‐1 Cells Treated With Spike Protein (S3) or Sars‐Cov‐2

**FIGURE 3:**
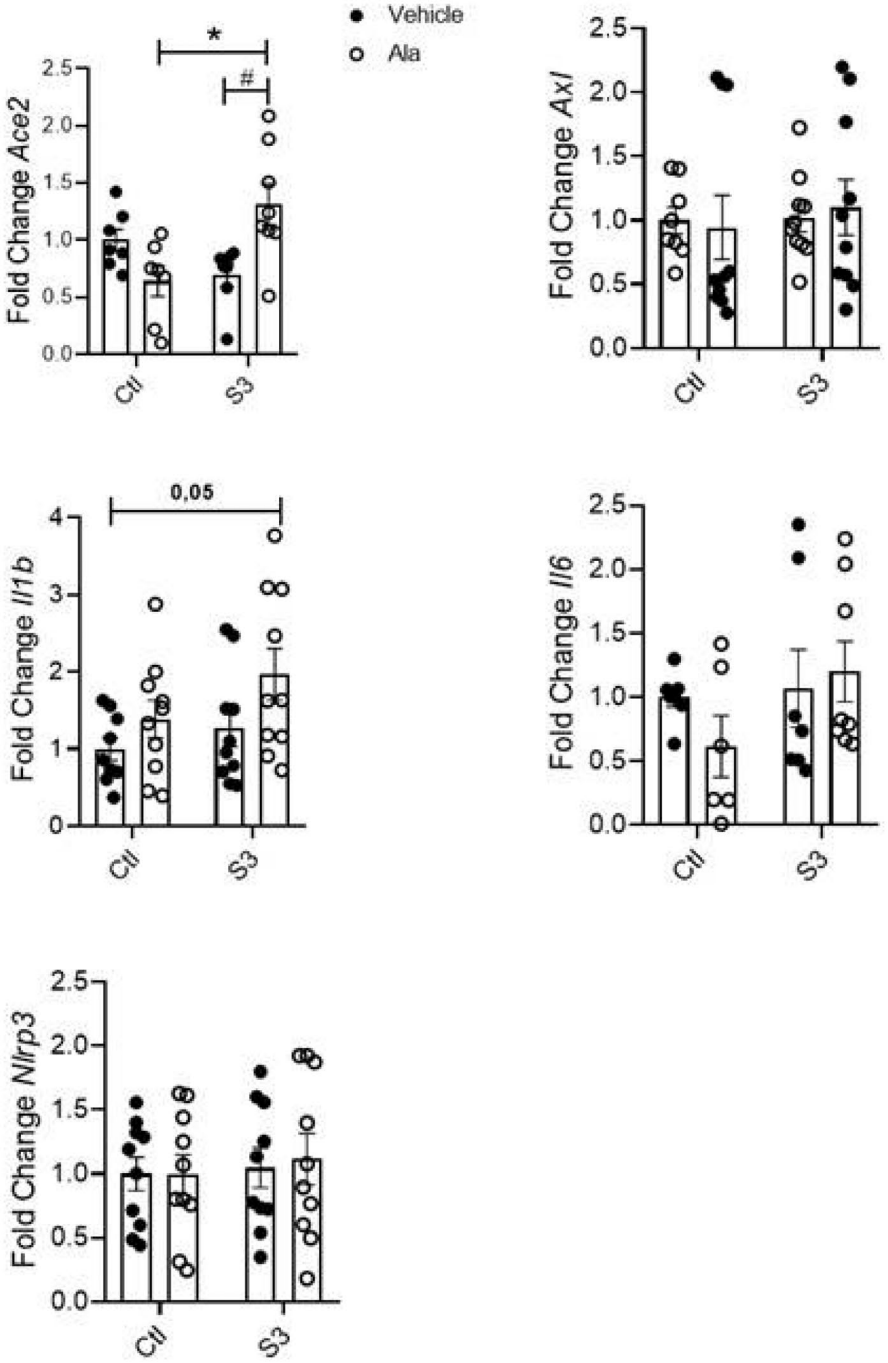
Gene expression in THP‐1 cells exposed to spike protein and Ala for 24 h. Data were compared with two‐way ANOVA followed by Tukey post‐test (*p < 0.05; #*p* < 0.05); *n* ≥ 3.

**FIGURE 4:**
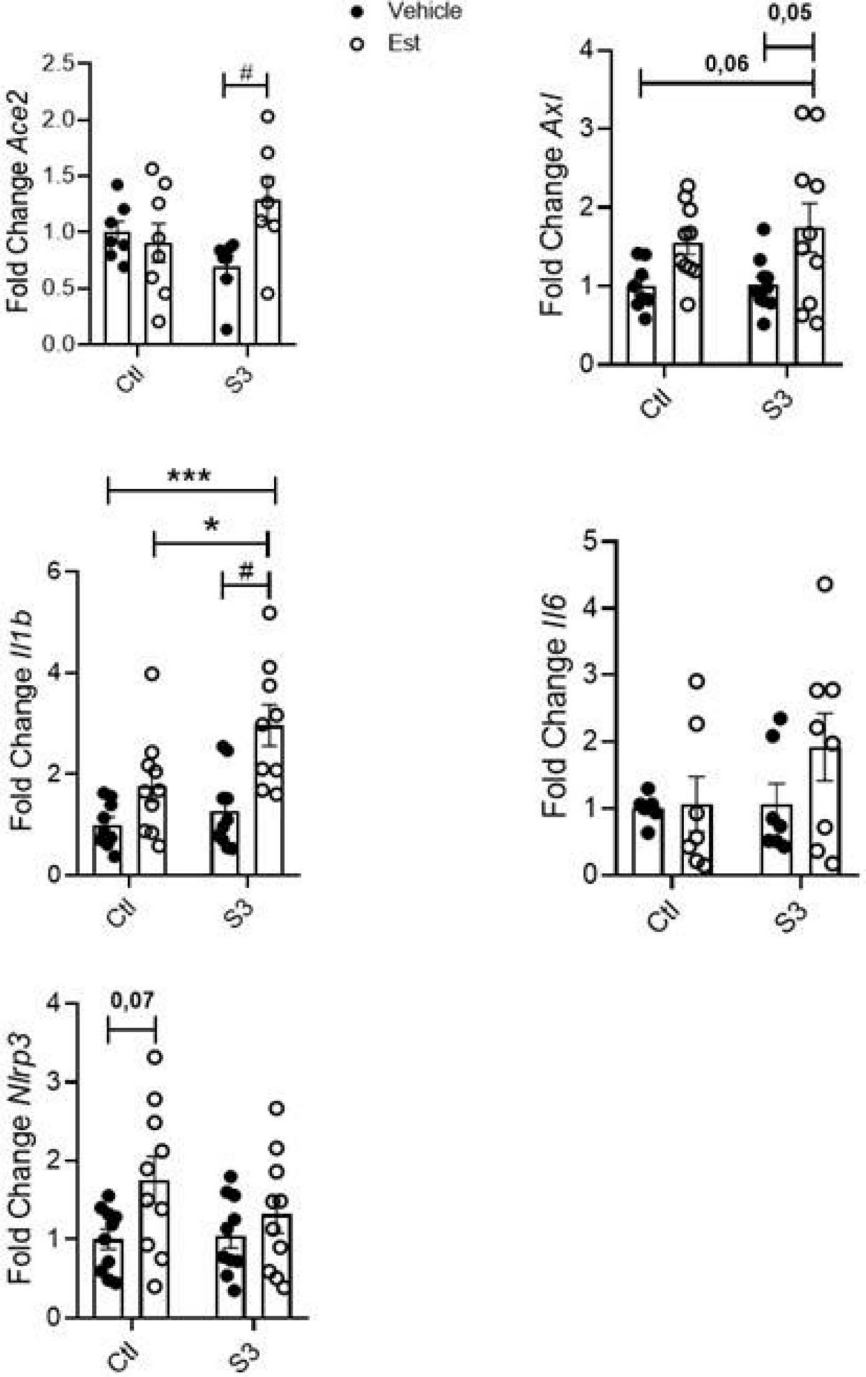
Gene expression in THP‐1 cells exposed to Spike protein and Est for 24 h. Data were compared with two‐way Anova followed by Tukey post‐test (*p < 0.05; ***p < 0.001; #*p* < 0.05); *n* ≥ 3.

Firstly, we performed experiments combining S3 with either Ala or Est in the culture medium of THP‐1 cells. We observed that Ala in combination with S3 was able to stimulate *Ace2* expression when compared to cells treated only with S3 or Ala. *Il‐1β* expression was increased in cells treated with the combination of S3 and Ala when compared to cells treated with vehicle only. Neither Ala nor S3 changed the expression of *Axl*, *Il‐6* and *Nlrp3* in THP‐1 cells (Figure 3). When Est was used in combination with S3, we noted a significant increase in *Ace2* and *Axl* expressions compared to cells exposed to S3 alone. The combination of Est and S3 was also able to stimulate *Il‐1β* expression when compared to the cells receiving either vehicle, Est or S3 (Figure 4).

**FIGURE 5:**
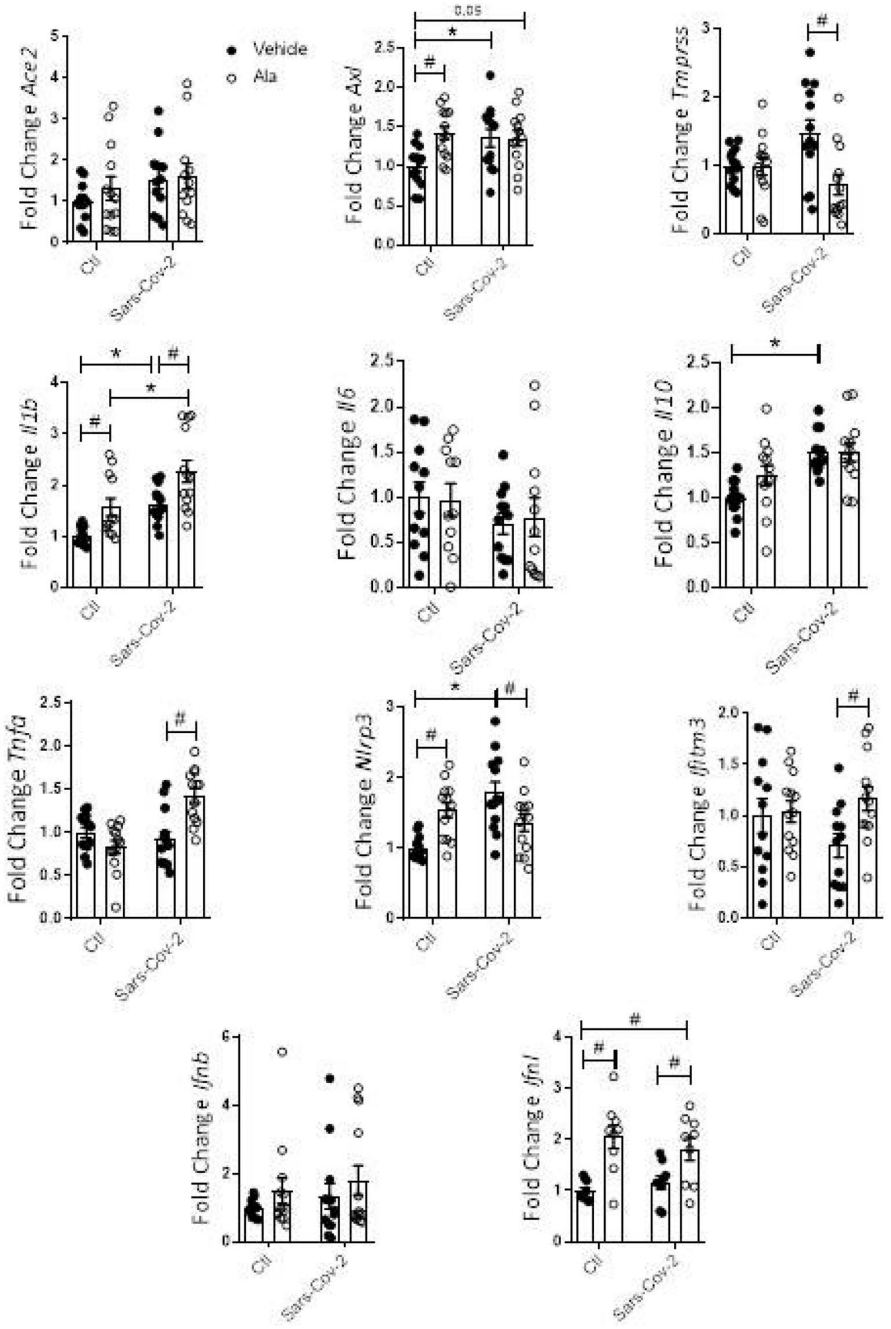
Gene expression in THP‐1 cells exposed to Sars‐Cov‐2 virus and Ala for 24 h. Data were compared with two‐way Anova followed by Tukey post‐test (*p < 0.05; #*p* < 0.05); *n* ≥ 3.

**FIGURE 6:**
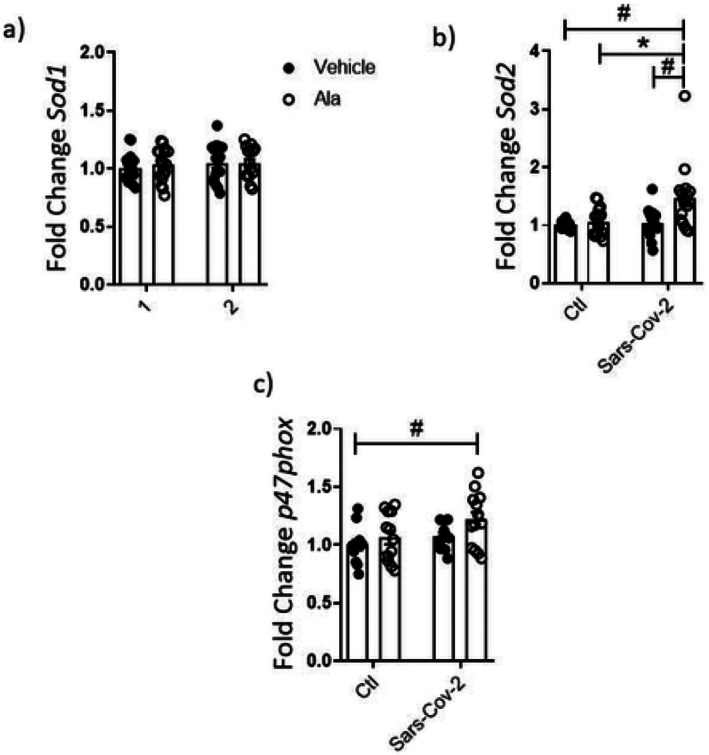
Gene expression in THP‐1 cells exposed to Sars‐Cov‐2 virus and Ala for 24 h. Data were compared with two‐way Anova followed by Tukey post‐test (*p < 0.05; #*p* < 0.05); *n* ≥ 3.

Next, the experiments were conducted in THP‐1 incubated with the Sars‐Cov‐2 virus and either Ala or Est. Ala by itself stimulated the expression of *Axl*, *Il‐1β*, *Nlrp3*, and *Ifnλ*. The sole exposure to Sars‐Cov‐2 instead stimulated the expression of *Axl*, *Il‐1β*, *Il10*, and *Nlrp3*. Sars‐Cov‐2 and Ala interacted to reduce *Tmprss* and *Nlrp3* and to further increase *Il‐1β*. On the other hand, the significant induction of *Il10* by Sars‐Cov‐2 was abolished by the combination with Ala. Interestingly, *Tnf‐a* and *Ifitm3* were stimulated in THP‐1 cells exclusively by the combination of Sars‐Cov‐2 with Ala (vs. cells exposed only to Sars‐Cov‐2) (Figure 5). Genes associated with the oxidant and antioxidant responses were also exclusively modulated by the combination of Ala and Sars‐Cov‐2. Only the combination of these two factors led to significant increases in *Sod2* and *p47phox* (Figure 6).

**FIGURE 7:**
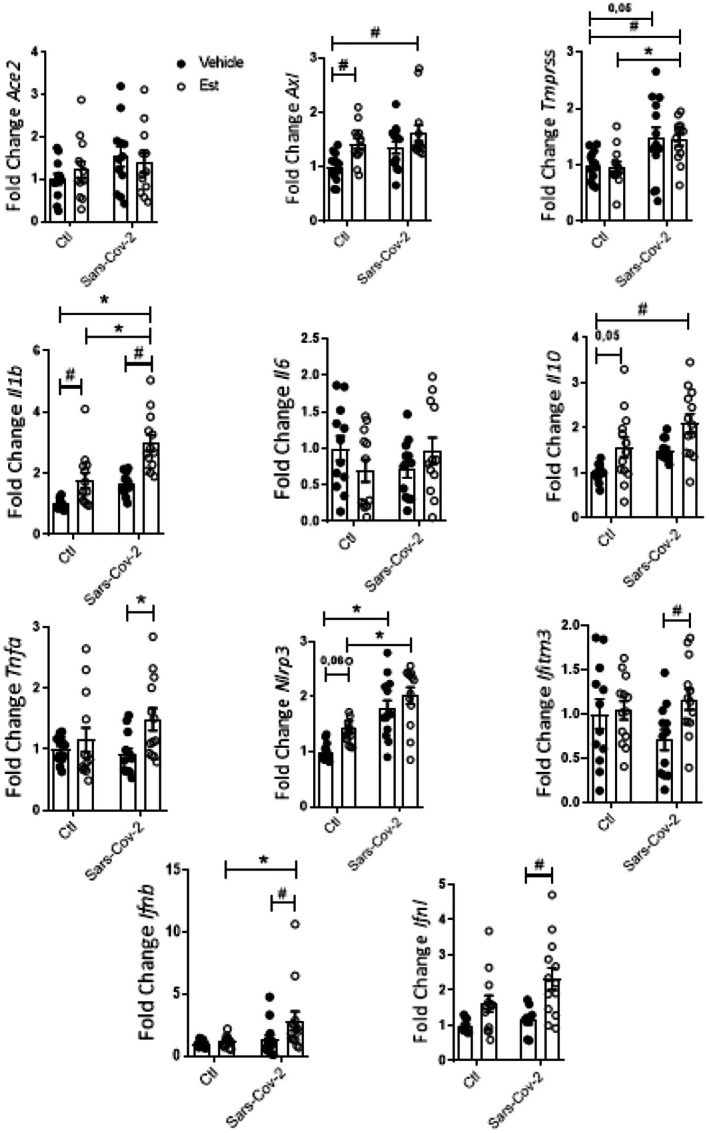
Gene expression in THP‐1 cells exposed to Sars‐Cov‐2 virus and Est for 24 h. Data were compared with two‐way Anova followed by Tukey post‐test (*p < 0.05; #*p* < 0.05); *n* ≥ 3.

**FIGURE 8:**
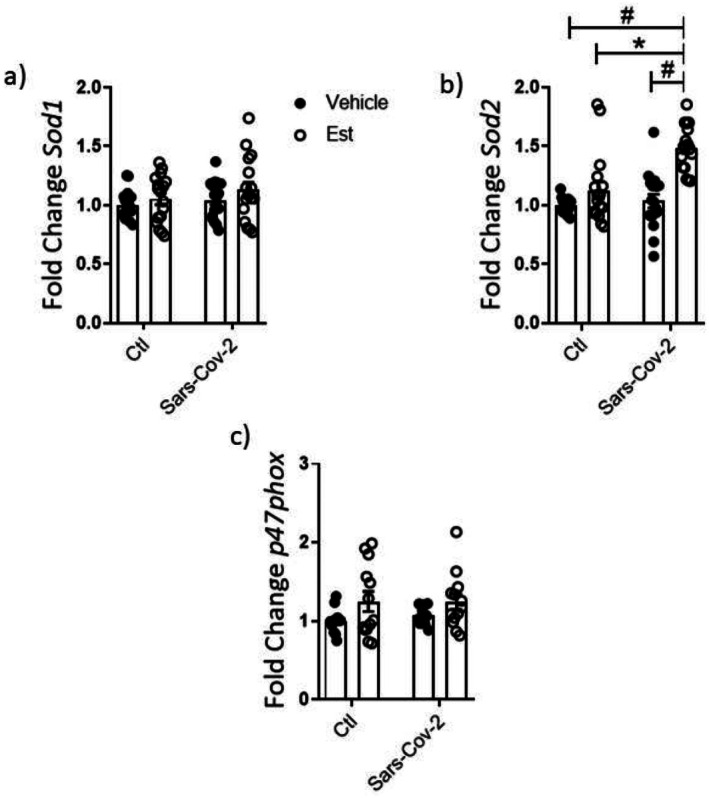
Gene expression in THP‐1 cells exposed to Sars‐Cov‐2 virus and Est for 24 h. Data were compared with two‐way Anova followed by Tukey post‐test (*p < 0.05; #*p* < 0.05); *n* ≥ 3.

THP‐1 cells treated with Est exhibited increased *Axl*, *Il‐1β* and *Il10* expression. Treatment with Sars‐Cov‐2 in the absence of Est increased *Tmprss* and *Nlrp3*. The combination of Sars‐Cov‐2 did not change the effects of Est over *Axl* and *Il10* expression but resulted in further increment of *Il‐1β*. Est, in turns, did not affect the modulation of Sars‐Cov‐2 over *Nlrp3*. Interestingly, the combination of Sars‐Cov‐2 and Est was the only experimental condition able to increase *Tnf‐a, Ifitm3, Ifnβ* and *Ifnλ* expressions (Figure 7). *Sod2* expression was modulated in THP‐1 derived macrophages treated with the combination of Sars‐Cov‐2 and Est (Figure 8).

## 4 Discussion

In the present study we describe yet unknown data showing that different types of fatty acid modulate the inflammatory response of macrophages to Sars‐Cov‐2. Our experiments were based on in vitro exposure of THP‐1 and Beas‐2b cells to Sars‐Cov‐2 yielding a nonproductive viral infection. Our in vitro approach was motivated by an in silico analysis of previously published single‐cell RNA sequencing transcriptome data revealing a key role of lung macrophages rather than alveolar epithelial cells in the inflammatory response of obese patients to Sars‐Cov‐2 (GSE171668). In general terms, we found that genes related to the inflammatory response as well as already described Sars‐Cov‐2 receptors exhibited a more evident modulation in lung macrophages than in alveolar epithelial cells. Moreover, when confronting data from COVID‐19 patients with different BMI, we also concluded that the genes related to the virus entry into the cells (*Tmprss2* and *Iftm3*) were increased in the lung macrophages of obese individuals.

Usually during viral infections, or in metabolic diseases like obesity, the macrophages are affected by chronic oxidative stress that reverberates in impaired immune function and cell death (Moreno‐Fernandez et al. 2022). Experimental evidence collected so far demonstrates a more intense inflammatory response when both viral infection and obesity are combined to each other. For instance, when infected with H1N1, mice rendered obese by high‐fat diet as well as leptin‐deficient obese mice have more evident lung damage and pulmonary edema as compared to lean controls (Smith et al. 2007; Zhang et al. 2013). In this way, our data provide evidence that helps to understand the mechanism underlying such metabolic/viral inflammatory interaction.

Aiming at clarifying the role of fatty acids in this interaction, we exposed THP‐1 derived macrophages to a combination of SARS‐Cov‐2 and stearic (Est) or alpha linolenic (Ala) acids. These fatty acids were chosen because they are described to modulate inflammatory response besides being highly elevated in obese patients. Ala, as a polyunsaturated fatty acid, could increase the expression of *Axl*, while Est, instead, increased the expression of *Axl* and *Tmprss2* when combined to Sars‐COV‐2. These two proteins are known to mediate Sars‐Cov‐2 entry to the cell (Cano et al. 2023). These data suggest that the increased expression of *Tmprss2* seen in lung macrophages of obese COVID patients might be caused by increased circulating free fatty acids commonly seen in individuals with high BMI. Curiously, when looking at the data from experiments with THP‐1, we also found that FFAs increase the expression of *Tnf‐α* and *Il‐1β* (Ala and Est) and *Il‐10*, *Ifnβ* and *Ifnα* (Est) when combined to Sars‐Cov‐2 (Baral et al. 2022; Nguyen et al. 2021).

The FFAs presently used were therefore able to increase the inflammatory response to the Sars‐Cov‐2 in THP‐1 derived macrophages in vitro. On the other hand, such inflammatory genes were not upregulated in lung macrophages of obese COVID‐19 patients. Our interpretation is that the changes in the internal milieu of an obese patient are complex and do not comprise an exclusive increase in circulating FFAs. The action of FFA in modulating Sars‐Cov‐2 induced changes in genes related to virus entry might prevail in vivo, while other factors related to obesity might overcome the effects of FFA on the inflammatory response. Interestingly, rodents rendered obese with HFD exhibit lower lung myeloperoxidase activity and reduced *Tnf‐α* and *Mcp‐1* expression in lung neutrophils after acute lung injury (ALI) induced by LPS (Maia et al. 2019). These findings are consistent with the phenomenon known as the “obesity paradox” in which obese patients with ALI or acute respiratory distress syndrome induced exhibit a higher survival rate than non‐obese patients (Ni et al. 2017). Although the precise mechanism underlying this phenomenon is not clarified, it was recently proposed that miRNA‐enriched exosomes from obese mice might attenuate lung inflammation induced by LPS (Wang, Zeng, et al. 2024).

In accordance to our data, the stearic acid (Est) is involved in the metabolic dysfunction‐associated steatotic liver disease (MASLD) that, in individuals infected with SARS‐CoV‐2, is related to *Ace2* upregulation and with a higher risk of liver injury (Cano et al. 2023). A recent study also showed the correlation of some non‐esterified polyunsaturated fatty acids (mainly linoleic and arachidonic acids) with a poor prognosis in COVID‐19 patients (Nguyen et al. 2021). As Ala is the precursor of two clinically important eicosanoids (eicosapentaenoic acid and docosahexaenoic acid) we speculate that the viral entry can be mitigated by modulating their content in the body (Baral et al. 2021).

Interestingly, our results showed that S3 combination to Ala or Est stimulated the expression of *Ace2* but only Est was able to boost *Il‐1β* expression in the presence of S3. This is consistent with previous findings showing that Ala, but not saturated fatty acids, interacts with spike protein to reduce viral infection (Staufer et al. 2022). The stearic fatty acid was already described to stimulate an inflammatory pathway, leading to an overexpression of *Il‐1β* in THP‐1 cells (Hung et al. 2023). In addition, it was previously described that stearic acid potentiated LDH‐a‐dependent production of lactate, which further stabilized HIF1α protein and increased *Vegf*, *Tnf‐a*, *Il‐1β*, and *Il‐6* in primary mouse chondrocytes (Miao et al. 2015). Although we have not addressed the precise mechanism by which FFA/Sars‐Cov‐2 interact to modulate virus entry to the cell, HIF1α can play a central role in this mechanism (Codo et al. 2020; Tian et al. 2021).

Our data also suggest that different parts of the Sars‐Cov‐2 other than S3 might interact with Ala to stimulate *Il‐1β* and *Tnf‐a*. In this context it is relevant to point out that the SARS‐CoV‐2 nucleocapsid protein is able to stimulate a widespread inflammatory response in lung epithelial cells leading to increase in multiple cytokines including TNFα (Wang, Tsai, et al. 2024). Whether Ala interacts with SARS‐CoV‐2 nucleocapsid protein however, is a matter that still requires further investigation.

Collectively, our data suggests a link between excess of NEFAs and severe COVID‐19 illness, contributing to the better understanding of recent epidemiological data showing that obesity is associated with poor prognosis for COVID‐19.

## Author Contributions

**Aline Rosa Maia:** data curation (equal), formal analysis (equal), methodology (equal), project administration (equal), validation (equal), writing – original draft (equal). **Bruna Rafaela dos Santos Silva:** data curation (equal), formal analysis (equal), investigation (equal), methodology (equal), writing – original draft (equal). **Pierina Lorencini Parise:** formal analysis (equal), methodology (equal). **Camila Lopes Simeoni:** formal analysis (equal), methodology (equal). **Luana Satelis Meira:** data curation (equal), methodology (equal). **José Luiz Proença Módena:** conceptualization (equal), formal analysis (equal), funding acquisition (equal), investigation (equal), writing – original draft (equal), writing – review and editing (equal). **Eliana Pereira Araújo:** formal analysis (equal), investigation (equal), writing – original draft (equal), writing – review and editing (equal). **Licio Augusto Velloso:** funding acquisition (equal), investigation (equal), resources (equal), supervision (equal), writing – original draft (equal), writing – review and editing (equal). **Gabriel Forato Anhê:** conceptualization (equal), formal analysis (equal), investigation (equal), methodology (equal), resources (equal), supervision (equal), visualization (equal), writing – original draft (equal), writing – review and editing (equal). **Joseane Morari:** conceptualization (lead), data curation (equal), formal analysis (equal), investigation (lead), methodology (lead), project administration (lead), supervision (lead), validation (equal), visualization (equal), writing – original draft (lead), writing – review and editing (lead).

## Conflicts of Interest

The authors declare no conflicts of interest.

## Supporting information


**Figure S1** Cellular Viability Test (MTT assay) of THP‐1 cells exposed to Ala or Est for 24 h.

**Figure S2** Viral quantification of SARS‐Cov‐2 using real‐time PCR.

Tables S1‐S2.

Tables S3‐S4.

Data S1.

## Data Availability

The data that support the findings of this study are available from the corresponding author upon reasonable request.
