# Supplementary figures and images for: Alpha Linolenic and Stearic Acids Modulate Genes Related to Viral Entry and Inflammatory Response in THP‐1 Derived Macrophages Exposed to SARS‐CoV‐2
Source: Food Sci Nutr. 2025 Sep 26;13(10):e70529. doi: 10.1002/fsn3.70529 (PMC12464564; doi:10.1002/fsn3.70529)

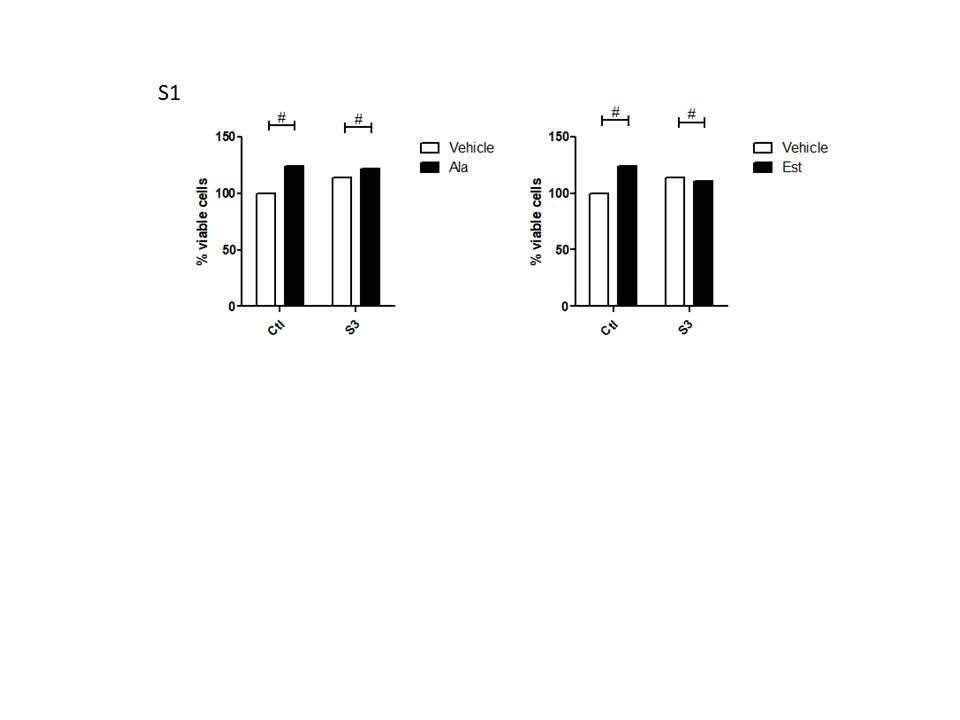

Supplement: Supplementary file 1 — Figure S1 Cellular Viability Test (MTT assay) of THP‐1 cells exposed to Ala or Est for 24 h. [file FSN3-13-e70529-s001.docx]

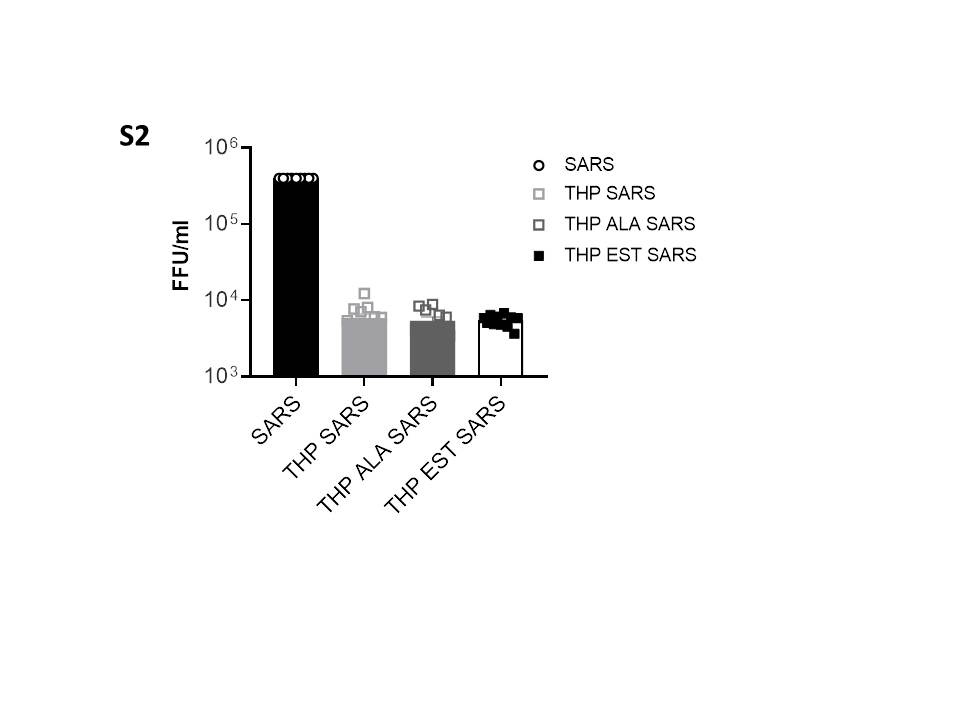

Supplement: Supplementary file 2 — Figure S2 Viral quantification of SARS‐Cov‐2 using real‐time PCR. [file FSN3-13-e70529-s003.docx]
