# Supplementary material for: Alpha Linolenic and Stearic Acids Modulate Genes Related to Viral Entry and Inflammatory Response in THP‐1 Derived Macrophages Exposed to SARS‐CoV‐2
Source: Food Sci Nutr. 2025 Sep 26;13(10):e70529. doi: 10.1002/fsn3.70529 (PMC12464564; doi:10.1002/fsn3.70529)
Supplement: Supplementary file 3 — Tables S1‐S2. [file FSN3-13-e70529-s005.docx]

Table S1. Assays used in qPCR.

| **Gene Name** | **Assay IDT DNA Technologies** | **Ref Seq** | **Exon Boundary** |
| --- | --- | --- | --- |
| *Ace2* | Hs.PT.58.27645939 | NM_021804(1) | 14-15 |
| *Axl* | Hs.PT.56a.19269785 | NM_001699(2) | 15-17 |
| *Il1β* | Hs.PT.58.1518186 | NM_000576(1) | 1-3 |
| **Gene Name** | **Assay Thermo Fisher** | **Ref Seq** | **Exon Boundary** |
| *Casp8* | Hs01594281_m1 | NM_001137667.1 | 8-9 |
| *Il10* | Hs00961622_m1 | NM_000572.2 | 4-5 |
| *Il6* | Hs00174131_m1 | NM_000600.4 | 3-4 |
| *Ifnβ* | Hs01077958_s1 | NM_002176.3 | 1-1 |
| *Ifnλ* | Hs00601677_g1 | NM_172140.1 | 1-2 |
| *Ifng* | Hs00989291_m1 | NM_000619.2 | 3-4 |
| *Ifitm3* | Hs03057129_s1 | NM_021034.2 | 2-2 |
| *Nlrp3* | Hs00918082_m1 | NM_001079821.2 | 3-4 |
| *Tmprss2* | Hs05024838_m1 | NM_001135099.1 | 4-5 |
| *Tnfα* | Hs00174128_m1 | NM_000594.3 | 3-4 |
| *Ppia* | Hs04194521_s1 | NM_001300981.1 | 6-6 |

Table with the name of the genes, the assays code , the reference sequence and region used to construct the assays. The assays were purchased from Thermo Fischer Scientific or IDT DNA Technologies.

Table S2. Primers used in qPCR (SYBR)

| **Gene Name** | **Ref. Seq** | **Forward (5’→3’)**  **Reverse (5’→3’)** | **Amplicon** | **Ref.** |
| --- | --- | --- | --- | --- |
| *Ccl22* | NM_002990.5 | gcgtggtgttgctaaccttca  aaggccacggtcatcagagt | 119 | 1 |
| *Cxcl10* | NM_001565.4 | gtggcattcaaggagtacctc  tgatggccttcgattctggatt | 198 | 2 |
| *Cxcl11* | NM_005409.5 | gacgctgtctttgcataggc  ggatttaggcatcgttgtccttt | 148 | 2 |
| *Gapdh* | NM_002046.7 | caacagcgacacccactcct  caccctgttgctgtagccaaa | 115 | 3 |
| *Mmr* | NM_002438.4 | gtggccggagtagtcatcat  tcttgaggtaggtgcacacg | 101 | 4 |
| *p47phox* | NM_000265.6 | agtcctgacgagacggaaga  ggacgggaagtagcctgtga | 189 | 5 |
| *Sod1* | NM_000454.5 | ctgaaggcctgcatggattc  ccaagtctccaacatgcctctc | 138 | 6 |
| *Sod2* | NM_000636.4 | ggcctacgtgaacaacctgaa  ctgtaacatctcccttggcca | 71 | 7 |

Table with the name of the genes, the reference sequence used to construct the primers, the primers sequence and the primers amplicon lenght. The primers were purchased from Exxtend.

**References**

1) L.E. Hind, E.B. Lurier, M. Dembo, K.L. Spiller, D.A. Hammer, Effect of M1–M2 Polarization on the Motility and Traction Stresses of Primary Human Macrophages, Cellular and Molecular Bioengineering. 9 (2016) 455–465. https://doi.org/10.1007/s12195-016-0435-x.

2) R.P. Hobbs, D.J. DePianto, J.T. Jacob, M.C. Han, B.-M. Chung, A.S. Batazzi, B.G. Poll, Y. Guo, J. Han, S. Ong, W. Zheng, J.M. Taube, D. Čiháková, F. Wan, P.A. Coulombe, Keratin-dependent regulation of Aire and gene expression in skin tumor keratinocytes, Nature Genetics. 47 (2015) 933–938. https://doi.org/10.1038/ng.3355.

3) M.B. Maeß, S. Sendelbach, S. Lorkowski, Selection of reliable reference genes during THP-1 monocyte differentiation into macrophages, BMC Molecular Biology. 11 (2010). <https://doi.org/10.1186/1471-2199-11-90>.

4) S. Hirakawa, Y.-K. Hong, N. Harvey, V. Schacht, K. Matsuda, T. Libermann, M. Detmar, Identification of Vascular Lineage-Specific Genes by Transcriptional Profiling of Isolated Blood Vascular and Lymphatic Endothelial Cells, The American Journal of Pathology. 162 (2003) 575–586. <https://doi.org/10.1016/S0002-9440(10)63851-5>.

5) S. Pendyala, I.A. Gorshkova, P. v. Usatyuk, D. He, A. Pennathur, J.D. Lambeth, V.J. Thannickal, V. Natarajan, Role of Nox4 and Nox2 in Hyperoxia-Induced Reactive Oxygen Species Generation and Migration of Human Lung Endothelial Cells, Antioxidants & Redox Signaling. 11 (2009) 747–764. <https://doi.org/10.1089/ars.2008.2203>.

6) T.-C. Hour, Y.-L. Lai, C.-I. Kuan, C.-K. Chou, J.-M. Wang, H.-Y. Tu, H.-T. Hu, C.-S. Lin, W.-J. Wu, Y.-S. Pu, E. Sterneck, A.-M. Huang, Transcriptional up-regulation of SOD1 by CEBPD: A potential target for cisplatin resistant human urothelial carcinoma cells, Biochemical Pharmacology. 80 (2010) 325–334. <https://doi.org/10.1016/j.bcp.2010.04.007>.

7) M. Hitchler, K. Wikainapakul, L. Yu, K. Powers, W. Attatippaholkun, F. Domann, Epigenetic Regulation of Manganese Superoxide Dismutase Expression in Human Breast Cancer Cells, Epigenetics. 1 (2006) 163–171. https://doi.org/10.4161/epi.1.4.3401.
