# Supplementary material for: Alpha Linolenic and Stearic Acids Modulate Genes Related to Viral Entry and Inflammatory Response in THP‐1 Derived Macrophages Exposed to SARS‐CoV‐2
Source: Food Sci Nutr. 2025 Sep 26;13(10):e70529. doi: 10.1002/fsn3.70529 (PMC12464564; doi:10.1002/fsn3.70529)
Supplement: Supplementary file 4 — Tables S3‐S4. [file FSN3-13-e70529-s002.docx]

**Table S3.** Demographic characteristics of the study population

| **Characteristics** | **Percentage (N)** |
| --- | --- |
| **Age** |  |
| 0-19 | 0.0% |
| 20-59 | 27.6% (8) |
| Above 60 | 72.4% (21) |
| **BMI** |  |
| Eutrophic | 10.3% (3) |
| Overweight | 31.0% (9) |
| Obese | 48.3% (14) |
| Unknown | 10.3% (3) |
| **Sex** |  |
| Females | 38.0% (11) |
| Males | 62.0% (18) |
| **Pre-existing condition** |  |
| Diabetes | 58.6% (17) |
| Hypertension | 86.2% (25) |
| Kidney disease | 27.6% (8) |
| Lung disease | 31.0% (9) |

Eutrophic, Overweight and Obese patients were classified according Body Mass Index (BMI) values respectivelly : 18.5-24.9, 25.0-29.9,>30.0. Kidney disease was defined as the presence of chronic kidney disease or baseline creatinine >1.5 or end-stage renal disease. Lung disease was defined as the presence of asthma or chronic obstructive pulmonary disease (COPD) or requiring home O_2_ or any chronic lung condition.

**Table S4.** Demographic characteristics of COVID patients according to their BMI status.

| **Characteristics** | **Eutrophic/Overweight**  **Percentage (N)** | **Obese**  **Percentage (N)** |
| --- | --- | --- |
| **Age** |  |  |
| 0-19 | 0.0% | 0.0% |
| 20-59 | 11.5% (3) | 15.4% (4) |
| Above 60 | 34.6% (9) | 38.5% (10) |
| **Sex** |  |  |
| Females | 11.5% (3) | 23.1% (6) |
| Males | 34.6% (9) | 30.8% (8) |
| **Pre-existing condition** |  |  |
| Diabetes | 26.9% (7) | 34.6% (9) |
| Hypertension | 42.3% (11) | 42.3% (11) |
| Kidney disease | 23.1% (6) | 7.7% (2) |
| Lung disease | 19.2% (5) | 11.5% (3) |

Eutrophic, Overweight and Obese patients were classified according Body Mass Index (BMI) values respectivelly : 18.5-24.9, 25.0-29.9,>30.0. Kidney disease was defined as the presence of chronic kidney disease or baseline creatinine >1.5 or end-stage renal disease. Lung disease was defined as the presence of asthma or chronic obstructive pulmonary disease (COPD) or requiring home O_2_ or any chronic lung condition.
