# Supplementary material for: Alpha Linolenic and Stearic Acids Modulate Genes Related to Viral Entry and Inflammatory Response in THP‐1 Derived Macrophages Exposed to SARS‐CoV‐2
Source: Food Sci Nutr. 2025 Sep 26;13(10):e70529. doi: 10.1002/fsn3.70529 (PMC12464564; doi:10.1002/fsn3.70529)
Supplement: Supplementary file 5 — Data S1. [file FSN3-13-e70529-s004.docx]

**Supplementary**

**Quantitative PCR**

All primers used in the study were certified for efficiency and specificity, as declared by the manufacturers. Nevertheless, we further validated the primers by amplifying the cDNA of each sample in triplicates at six different concentrations (3-fold serial dilutions). Both the primers for the target genes and reference gene were tested. The efficiency of the system was calculated using the formula: E = 10 (-1 / slope) – 1.
